# Gender and ethnicity's influence on first‐year nursing students' educational motivation and career expectations: A cross‐sectional study

**DOI:** 10.1002/nop2.1191

**Published:** 2022-02-11

**Authors:** Sigurd Maurud, Elin Børøsund, Anne Moen

**Affiliations:** ^1^ Department of nursing science University of Oslo Oslo Norway; ^2^ 155272 Department of Digital Health Research Oslo University Hospital Oslo Norway

**Keywords:** ethnicity, expectations, gender, motivation, nursing, students

## Abstract

**Aim:**

Given the apparent link between gender and ethnicity, and the diversity and career opportunities in nursing, this study examined gender and ethnicity's influence on first‐year nursing students' educational motivation and career expectations.

**Design:**

Cross‐sectional.

**Methods:**

Through bootstrapped linear regressions, we analysed data on 504 Norwegian first‐year nursing students' self‐reported educational motivation and career expectations, from the StudData survey at the Centre for the Study of Professions (SPS) at Oslo Metropolitan University (OsloMet).

**Results:**

The sample consisted of 67 (13%) male and 437 (87%) female nursing students. Female students were more motivated compared to male students by professional interest and to pursue a specialization, less likely to assume leadership positions in the future, and more likely to prioritize family and pursue positions in the traditional nursing field. In total, 425 (84%) respondents stated a Norwegian background. Respondents who stated that both of their parents were born in a country other than Norway made up the 79 (16%) students of immigrant background. Those with immigrant backgrounds were more motivated than other students by income, status and flexible working hours and less likely to pursue a specialization or future employment in the nursing field.

## 1 INTRODUCTION

According to the World Health Organization (WHO), there was a global nursing shortage of 5.9 million nurses worldwide in 2018 (WHO, 2020). In addition, many countries have an ageing nurse workforce, and there is a need to increase efforts in recruiting and retaining of nurse graduates. Nursing is a highly mono‐gendered profession. Approximately 90% of the world's nursing workforce is female (WHO, 2020). In many countries, men make up a small, stable and significantly small proportion of the nurse workforce, with around 11% in the United States (Auerbach et al., 2017) and the United Kingdom (Williams, 2017). In the Scandinavian countries the proportions are 3.2% in Denmark (Søndergaard, 2008), 12% in Sweden (Socialstyrelsen, 2019) and 10% in Norway (Statistics Norway [SSB], 2020a). Many nurses start their careers in somatic nursing; however, compared to female nurses, more male nurses seem to pursue nursing positions in psychiatry. The male nurses are also more likely to assume leadership positions, or to take positions outside of traditional nursing and health care, such as in companies and industrial firms (Abrahamsen, 2004).

Adding to the recruitment and retention concerns, people with immigrant backgrounds generally have a lower probability of employment in occupations that require professional authorization, such as nursing, than people without immigrant backgrounds (Drange & Alecu, 2019). In addition, nurses with immigrant backgrounds are more likely to work in elderly care than other nurses (Drange & Karlsen, 2016).

Nursing is a profession that deals with matters of health, illness, life and death. The nursing shortage is critical and there is a widely acknowledged need to increase nursing graduates worldwide (WHO, 2020). Gender segregation can lead to loss of diversity and talent, and constitutes one of the main contributions to uneven workforce capacities across occupations, maintaining inequality and reinforcing gender stereotypes (WHO, 2019). The COVID‐19 pandemic has demonstrated the importance of measures to fight systemic inequalities, systemic biases and systemic racism (Bhatia, 2020). Furthermore, a sufficiently diverse nursing workforce is more likely to support all patients and people in need of care, and hence acknowledge the importance of ethnicity and respect for cultural diversity (American Association of Colleges of Nursing, 2019). The Norwegian authorities seek to equalize the distribution of men and women in educational fields that are dominated by one gender (Ministry of Children, Equality & Family, 2015). Moreover, promoting equal labour market participation opportunities, regardless of gender and ethnicity, is an explicit and reiterated goal of policy makers (The Royal Norwegian Ministry of Finance, 2019). Despite general attention on promoting equal opportunities and explicit policy support, the nursing profession is still largely mono‐gendered. Therefore, there is still a need to explore and better understand the influence of gender and ethnicity on why individuals choose their education, where and with whom they expect to work. By examining nursing students' educational motivation for selecting nursing and what their career expectations are at entry to the education, the present study aimed to gain further insights into how gender and ethnicity differences affect choices to join the nursing profession.

## 2 BACKGROUND

Previous studies have indicated possible gender‐ and age‐related differences among students, and ethnicity seems to influence the choice to pursue higher education. In nursing education, men appear to be older than women when they start their studies in this field and male nursing students typically perceive themselves as competent leaders to a greater degree than females (Hoffart et al., 2019). Female nursing students appear to have stronger family‐related values than male nursing students (Ten Hoeve et al., 2016), and others consider females more suitable for the nursing profession than males (Zysberg & Berry, 2005).

Meanwhile, compared to young Norwegians without immigrant backgrounds, young Norwegians with immigrant backgrounds have expressed stronger orientation toward higher education (Bakken & Hyggen, 2018). Previous research has also identified differences and similarities in the educational motivations and career orientations of male and female nursing students and of nursing students with and without immigrant backgrounds.

### 2.1 Motivation

Motivation is crucial in order for people to work toward a task and is a phenomenon concerned about the amount of motivation as well as the underlying goals and attitudes that provide the orientation of the motivation. Studies often make a distinction between intrinsic and extrinsic motivation when describing what drives an individual to action (Ryan & Deci, 2000). Intrinsic motivation describes activities that directly give satisfaction to the individual. Extrinsic motivation is driven by external factors, such as the possibility of achieving a higher occupational status, a higher income and other occupational benefits (Ryan & Deci, 2000).

Traditionally, the educational motivation of nursing students has been the opportunity to help others (Dæhlen, 2003). Most people have the ability to experience selflessness, and this form of motivation can be described as an altruistically driven intrinsic motivation (Nortvedt, 2012). However, previous studies of the altruistic educational motivation of male and female nursing students do not show unambiguous, conclusive results. One study found strong altruistic educational motivation in both male and female nursing students (Harding et al., 2018). In other studies, female nursing students displayed a stronger altruistic motivation than male nursing students (Hoffart et al., 2019). Meanwhile, male nursing students seem to be more concerned with income and job security than female nursing students (Zysberg & Berry, 2005).

Little is known about the ethnic background and educational motivation of nursing students. However, Basit (2012) found that young people with immigrant backgrounds are more driven to pursue higher education because of the opportunity it offers for a better socioeconomically status than other students. Meanwhile, Abrahamsen and Drange (2014) showed that compared with students without immigrant background, students with Asian immigrant backgrounds had a higher extrinsic educational and that income and opportunities for promotions were central to their choice of pursuing higher education.

### 2.2 Career

In the context of the present study, the term career refers to an individual's professional development, mobility and personal capacities as well as opportunities for choices and actions that set the conditions for his/her transition to new positions (Hughes, 1997). There are several categories of career mobility, and its categories are based on whether the job status, employer or function changes (Ng et al., 2007). In the nursing profession, career mobility can be vertical or horizontal. Horizontal mobility describes work changes at the same hierarchical level within a single organization or across employers. Employees may change jobs, employer and setting, but retain the same job title, job status, and/or work tasks. Vertical career mobility is defined as an upward or downward transition to a job with a different hierarchical ranking, such as a transition to a leadership position (Ng et al., 2007).

Nursing is offered to many different patient groups, giving nurses a wide variety of possible jobs and settings, fields of study, and specializations (Norwegian Directorate of Health, 2017). Positions in child health and surgery appear to be the most sought‐after among nursing students (Hunt et al., 2020; Kloster et al., 2007). These specializations provide contact with children, address preventive health, and/or emphasize technical and therapeutic competence (Kloster et al., 2007). Such career choices can be characterized as forms of horizontal and vertical mobility. For example, a master's degree is needed to work as a midwife in Norway (Norwegian Directorate of Health, 2017), and this degree will also somewhat increase a nurse's income. The least sought‐after positions by nursing students are in elderly care (Hunt et al., 2020; Kloster et al., 2007).

Previous studies have indicated differences in male and female nursing students' career expectations. Among the many opportunities available for horizontal mobility in nursing, nursing students generally consider most of them to be best suited for women (McLaughlin et al., 2010; Muldoon & Reilly, 2003). Women may be viewed as more caring than men which may be reflected in selection of nursing areas where relational competence is important (Myklebust, 2020). Male nursing students have expressed higher expectations to work in leadership positions than female nursing students (Zysberg & Berry, 2005). They also have lower expectations of working as staff nurses after finishing their education than their female counterparts (Abrahamsen & Storvik, 2019). Male nursing students appear thus to be more oriented toward a vertical career progression than female nurses.

Just as we know little about the educational motivation of nursing students with immigrant backgrounds, we also have insufficient knowledge about their career expectations. It appears, however, that people with immigrant backgrounds often have different career expectations compare to those without immigrant background. Basit (2012) found that young people with immigrant backgrounds perceive education and occupation as ways to increase their socio‐economic status, gain a degree of independence and achieve better conditions so that they can support their families. Compared with other students, students with Asian immigrant backgrounds seems to be more driven to pursue a master's degree but less driven to be employed in a field related to their education or to pursue a leadership position (Abrahamsen & Drange, 2014).

Meanwhile, the global shortage of nurses (WHO, 2020), including the Norwegian labour market (Kalstø., 2019), demands more nurses than the labour market can access. This trend is expected to increase (Hjemås et al., 2019), and may in fact explain findings where nursing students, regardless of ethnicity, appear to be optimistic about opportunities for relevant paid work after their studies (Orupabo, 2014).

### 2.3 Theoretical framework

This study is based on theoretical approaches that seek to elaborate on gender and ethnicity respectively. Charles and Grusky (2004) address the segregation of men and women in the labour market by distinguishing between horizontal and vertical occupational segregation. There is segregation between men and women across the divide of manual and non‐manual occupations, as well as the most desirable positions in occupations on both sides of this divide. Vertical segregation is viewed as a product of the belief of male primacy. Even though gender‐egalitarian forces undermines this form of segregation, horizontal segregation is maintained and generated through social presumptions of essential female and male traits, and their coherence with male‐ and female‐dominated occupations (Charles & Bradley, 2009; Charles & Grusky, 2004). Of relevance to this study and as pointed out by England (2010), equal access to the labour market have led women to upward mobility through male‐typical occupations, but without challenging the devaluation of female‐typical occupations (England, 2010).

In addition to gender, this study also examined ethnicity's influence on first‐year nursing students' educational motivation and career expectations. Socialization can affect career choices of people based on their ethnicity. Modood (2004) links socialization to ethnic capital, where ethnicity contributes to a resource‐strengthening socialization among certain groups with immigrant backgrounds, which, in turn, leads to a higher educational motivation. However, ethnic capital can also be inhibiting. For example, cultural gender differences can limit an individual's career and education choices (Shah, 2007). Ethnic capital appears to both promote and inhibit educational achievements and is probably related to young people's experience of autonomy in relation to their parents (Leirvik, 2016).

Meanwhile, discriminatory hiring practices by employers may limit the opportunities of people with immigrant backgrounds in the labour market (Midtbøen & Rogstad, 2012). Not all have the necessary resources or capabilities to challenge such barriers; however, some people with immigrant backgrounds have developed strategies and behaviours to reduce the personal impact of these barriers (Modood & Khattab, 2016). Related to our study and pointed out by Lee and Zhou (2015) is for example the view of the pursuit of high education and competence levels as a strategy to counteract discriminatory employment practices (Lee & Zhou, 2015).

### 2.4 Research Question

The purpose of this study was to investigate whether nursing students' gender and ethnicity influences their motivation to pursue nursing education and their career expectations.

## 3 THE STUDY

### 3.1 Design

A cross‐sectional design was used to examine nursing students' motivation for pursuing nursing education and career expectations.

### 3.2 Methods

#### 3.2.1 Sample

The data analysed in the present study was from a 2012 StudData survey by the Centre for the Study of Professions (SPS) at Oslo Metropolitan University, Norway.^1^ StudData is a longitudinal panel database with data that enables investigation of factors, such as educational motivation; career expectations; attitudes; job values; educational and professional experiences; and the transition from education to work among students studying professional fields, such as teaching, engineering and nursing. The database includes four panels, and each panel consists of participants who have completed surveys in different phases of their education (SPS, 2005). The present study used data from first‐year nursing students in the survey's fourth panel, which focus on recruitment and motivation related to students' educational choices (SPS, 2005).

#### 3.2.2 Data collection

The survey data used in the present study were collected in fall 2012. All first‐year nursing students at three educational institutions, which were located in eastern, western and northern Norway, were asked to participate. The students were introduced to the survey in connection with their classes, and the StudData questionnaire^2^ was then delivered via e‐mail. Invitations were also sent to students who were not present at the classes. Out of 1,011 total nursing students, 526 answered the survey. The 22 respondents who did not state their gender or ethnicity were excluded from our analysis, leaving a sample population of 504 students and a 49.8% response rate.

### 3.3 Analysis

#### 3.3.1 Variables

##### Demographics: Gender, age and ethnicity

Table 1 shows the independent variables used in the analyses: *gender, age* and *ethnicity*.

**TABLE 1:** Demographic characteristics of the sample population (*N* = 504)

	*N*	%
Gender		
Men	67	13.3
Women	437	86.7
Age		
Under 24 years old	372	73.8
24 years old or older	132	26.2
Ethnicity		
Students with immigrant backgrounds	79	15.7
Other students	425	84.3

Most of the 504 respondents identified as female. Those that identified as male were used as reference group in the analyses. Student age was divided into two groups in the StudData set: under 24 years old and 24 years old or older. 35 (52.2%) males and 97 (22.2%) females were 24 years old or older in the survey. The under 24 years old group was used as a reference group in the analyses.

Ethnicity is a concept that is used to categorize individuals based on their place of birth and cultural background (Schackt, 2020). In our analyses, ethnicity was used as an independent variable (see Table 1) and was divided into two categories: “students with immigrant backgrounds” and “other students.” The former category included the 79 respondents who stated that both of their parents were born in a country other than Norway; within this category, 45 respondents were born abroad to two foreign‐born parents and 34 respondents were born in Norway to two foreign‐born parents. The rationale for including both these groups in a single category, and not include respondents with only one immigrant parent in the category, lies in the fact that the members of these groups have different backgrounds and are exposed to different cultural influence than the respondents without immigrant background. With the exception of Norway and the other Nordic countries, respondents only had the ability to state in which continent they and their parents were born. In total, 477 had a Norwegian or Nordic background, 30 had a non‐Nordic European background, 3 had a North American or Australian background, 59 had an Asian or African background and 2 had a South American or Central American background. The 425 respondents who stated that one or both of their parents were from Norway comprised the other students category. The students with immigrant backgrounds category were used as reference group in the analyses.

##### Educational motivation and career expectations

As shown in Table 2, the dependent variables examined four aspects of educational motivation and four aspects of career expectations. All survey questions and response options were translated into English for this paper by the authors. The variables that examined students' *educational motivation* were constructed as indexes comprised of statements that the students rated based on the following three survey questions: 1. “How important do you think the following factors are when evaluating a job?” (response options: 1 = very important to 5 = not important at all and 6 = don't know), 2. “To what extent have the following factors contributed to you choosing the education you are currently pursuing?” (response options: 1 = to a very large extent to 5 = not at all) and 3. “To what extent do the following statements apply to your choice of education?” (response options: 1=to a very large extent to 5=not at all and 6=irrelevant/don't know). The *altruism* and *professional interests* indexes examined students' intrinsic educational motivation, while the *income and status* and *anticipated time flexibility* indexes examined their extrinsic educational motivation. The associated variables for each question are shown in Table 2.

**TABLE 2:** Nursing students' average values for dependent variables related to educational motivation and career expectations

Indexes				
Type of motivation	Index	Variables	Mean (*SD*)	*α*
Extrinsic motivation	Income and status	High income^a^	2.90 (0.04)	0.55
		The education programme leads to jobs with a high reputation and status.^b^		
	Anticipated time flexibility	A job that provides a lot of free time^a^	3.46 (0.03)	0.60
		A job with flexible work hours^a^		
		Good opportunities to balance work with family obligations^a^		
		A job with an opportunity for part‐time work^a^		
Intrinsic motivation	Altruism	A job where one can help others^a^	4.61 (0.02)	0.69
		A job that is beneficial to society^a^		
	Professional interests	I have chosen this programme because I want to go into a specific profession.^c^	4.34 (0.02)	0.74
		I am interested in the profession^b^		
		Fits well with my talents/qualities^b^		
		It is interesting to study.^b^		
		An interesting job^a^		

Other dependent variables		
		Mean (*SD*)
Career expectations	I have a leadership position.^d^	3.70 (0.05)
	I have pursued a specialization.^d^	4.26 (0.05)
	I spend a great deal of time with my family.^d^	4.29 (0.04)
	I have a career in my current field of study ^d^	3.98 (0.05)

Abbreviations: *SD*, standard deviation; α, Cronbach's alpha.

^a^ Response options to “How important do you think the following factors are when evaluating a job?” on a five‐point scale from 1 = not important at all to 5 = very important.

^b^ Response options to “To what extent have the following factors contributed to you choosing the education you are currently pursuing?” on a five‐point scale from 1 = not at all to 5 = to a very large extent.

^c^ Response options to “To what extent do the following statements apply to your choice of education?” on a five‐point scale from 1 = not at all to 5 = to a very large extent.

^d^ Response options to “When you imagine your life in 10 years, how likely is it that the statements below would describe your situation?” on a five‐point scale from 1 = does not describe my situation at all to 5 = describes my situation very well.

The answer options were recoded from 1 = not important at all/not at all to 5 = very important/to a very large extent. The irrelevant/don't know answer option was merged with and coded as missing answers. The variables were then collected in indexes that measured the extrinsic and intrinsic motivational factors.

The variables related to *career expectations* corresponded to statements that the students rated based on the following question: 4. “When you imagine your life in 10 years, how likely is it that the statements below would describe your situation?” (see Table 2). The answers were measured on a five‐point scale (1 = fits very well to 5=does not fit at all). The associated variables for each question are shown in Table 2. The statement, “I work in in a field other than the one that I am currently studying,” was recoded to “I have a career in my current field of study,” and the answer options were recoded from 1 = does not describe my situation at all to 5=describes my situation very well.

#### 3.3.2 Data analysis

The data analyses were conducted using Statistical Package for the Social Sciences (SPSS) version 25 for Windows. We performed linear regressions of each of the motivation indexes and the variables dealing with career expectations. All independent variables were included in each dependent variable regression.

#### 3.3.3 Validity and reliability

This study used self‐reported data on educational motivation and career expectations, elicited from four questions in the StudData set. Most of the statements from question 1 were collected from an established instrument (ISSP Research Group, 1999). Statements from the other questions were developed by the group of researchers establishing StudData, to complemented established instruments, to elicit data that map students' educational motivation and career expectations.

We bootstrapped the regressions after controlling the variables for the ability to perform parametric analyses. Bootstrapping estimates a population's characteristics through random and repeated sampling of the data set (Skovlund, 2019). In our analyses, we used 1,000 bootstrap samplings for each regression (Field, 2018). The dependent variables comprise items from a survey relevant for this study, and do not constitute established construct measures of educational motivation and career expectations. To assess the suitability of the variables for index construction, correlation between the variables of the indexes was assessed and Cronbach's alpha values were calculated for each index with the lower limit set at 0.55 (see Table 2). The variables related to career expectations did not meet the criteria for constructing an index and were therefore treated as separate dependent variables in the analyses.

### 3.4 Ethical considerations

In accordance with local regulations, a data processor agreement regarding the use of material from StudData and containing a declaration of confidentiality was signed with SPS, OsloMet and University of Oslo (SPS, 2018). The data were anonymized.

## 4 RESULTS

The results depict our analyses of four factors related to educational motivation and four factors related to career expectations.

### 4.1 Nursing students' educational motivation and career expectations

Table 2 shows an overview of the dependent variables' results, including their mean values. The indexes measuring intrinsic motivation had a higher mean than those measuring extrinsic motivation. The *altruism* and *professional interests* indexes had the highest means. The possibility of flexible work hours seemed somewhat important, as expressed through the *anticipated time flexibility* index, while *income and status* were the students' least important factors related to educational motivation. The students' greatest expectations concerned spending time with family. They also expressed high expectations of specializing in nursing and working as nurses in the future. The students' lowest expectations were related to leadership positions.

### 4.2 Associations between gender and ethnicity and nursing students' educational motivation and career expectations

Table 3 shows the results of the regressions of the four motivational factors for pursuing nursing education. Age had a statistically significant association with one of the educational motivation factors. Specifically, students who were 24 years old or older had a significantly lower educational motivation in terms of *income and status*, compared to younger students (B = −0.21, *p <* .05). Gender had a statistically significant association with one motivational factor. Specifically, women reported a significantly higher motivation than men related to *professional interests* (B = 0.16, *p* < .05). Ethnicity seemed to be important for students' educational motivation. Nursing students with immigrant backgrounds had a statistically significant higher educational motivation in terms of *income and status* (B = −0.46, *p* < .01) and *anticipated time flexibility* (B = −0.18, *p* = .05).

**TABLE 3:** Linear regressions on the influence of gender, ethnicity and age on nursing students' educational motivation

	Intrinsic motivation						Extrinsic motivation					
	Altruism^a^ (*n =* 495)			Professional interests^b^ (*n* = 482)			Income and status^c^ (*n =* 478)			Anticipated time flexibility^d^ (*n =* 465)		
	*B*	95% CI	*p*	*B*	95% CI	*p*	*B*	95% CI	*p*	*B*	95% CI	*p*
Gender (ref. = men)	0.005	−0.13, 0.14	.946	0.16	−0.01, 0.33	.04	−0.025	−0.25, 0.20	.80	0.062	−0.12, 0.25	.51
Age (ref. = under 24 years old)	−0.025	−0.13, 0.08	.653	0.11	−0.01, 0.21	.06	−0.21	−0.40, −0.01	.03	−0.013	−0.16, 0.13	.87
Ethnicity (ref. = students with immigrant backgrounds)	−0.10	−0.23, 0.02	.111	0.10	−0.06, 0.28	.23	−0.46	−0.69, −0.21	.001	−0.18	−0.35, −0.001	.05

Abbreviations: 95% CI, bias corrected, and accelerated confidence interval based on 1,000 bootstrap samplings; B, regression coefficient; *p*, *p*‐value.

^a^ $R_{\text{adj}}^{2}$ = −0.001 (*p* = .463).

^b^ $R_{\text{adj}}^{2}$ = 0.010 (*p* = .047).

^c^ $R_{\text{adj}}^{2}$ = 0.036 (*p* < .001).

^d^ $R_{\text{adj}}^{2}$ = 0.003 (*p* = .231).

Table 4 shows the results of the regressions of the four career expectations. Age had no significant association to the students' career expectations. Regarding gender, compared to male nursing students, female students had significantly lower expectations of leadership positions (B = −0.34, *p* < .05). However, female students had higher expectations of spending time with family (B = 0.6, *p* < .01) and working as nurses (B = 0.36, *p* < .05) than male students. Female students also had somewhat higher expectations of pursuing a specialization than male students; however, the difference was not significant. Ethnicity also impacted the nursing students' career expectations. Compared with other students, students with immigrant backgrounds had significantly lower expectations of pursuing a specialization (B = 0.53, *p <* .01) and working as nurses (B = 0.39, *p* < .05).

**TABLE 4:** Linear regressions on the influence of gender, ethnicity and age on nursing students' careers and family life expectations in 10 years

	Leadership position^a^ (*n* = 497)			Specialization^b^ (*n* = 498)			Spending a lot of time with family^c^ (*n* = 499)			Working as a nurse^d^ (*n* = 500)		
	*B*	95% CI	*p*	*B*	95% CI	*p*	*B*	95% CI	*p*	*B*	95% CI	*p*
Gender (ref. = men)	−0.35	−0.61, −0.07	.012	0.32	0.01, 0.63	.063	0.59	0.36, 0.84	.001	0.39	0.06, 0.70	.016
Age (ref. = under 24 years old)	0.06	−0.19, 0.30	.615	−0.10	−0.34, 0.17	.399	0.13	−0.03, 0.29	.12	0.20	−0.07, 0.45	.12
Ethnicity (ref. = students with immigrant backgrounds)	−0.17	−0.43, 0.06	.191	0.54	0.20, 0.87	.002	0.13	−0.09, 0.34	.24	0.41	0.10, 0.75	.014

Abbreviations: 95% CI, bias corrected, and accelerated confidence interval based on 1,000 bootstrap samplings; B, regression coefficient; *p*, *p*‐value.

^a^ $R_{\text{adj}}^{2}$ = 0.015 (*p* = .014).

^b^ $R_{\text{adj}}^{2}$ = 0.044 (*p* < .001).

^c^ $R_{\text{adj}}^{2}$ = 0.056 (*p* < .001).

^d^ $R_{\text{adj}}^{2}$ = 0.024 (*p* = .002).

## 5 DISCUSSION

This study examined the influence of gender and ethnicity on nursing students' educational motivation and career expectations. We analysed four factors related to educational motivation and four factors related to career expectations.

### 5.1 Educational motivation

The present study's findings indicated few differences between male and female students' educational motivation. Some of the results contrast reports in previous studies, which showed that men are more driven by income, job security and occupational status (Zysberg & Berry, 2005), while women are more driven by altruism (Hoffart et al., 2019). There was little evidence in this study that men are more driven than women in terms of income and occupational status, and the findings are in line with Harding et al. (2018), that showed that both men and women were motivated by altruism. In addition, our findings did not show any significant differences between male and female students in terms of the *anticipated time flexibility* motivational factor. However, female students had significantly higher motivation in terms of the *professional interests* factor than male students.

Based on the correspondence between traits regarded as distinctively male or female and the task requirements of male‐ and female‐dominated careers, respectively, Charles and Grusky (2004) claims that the tendency for men and women to enter gender‐typical occupations is maintained through presumptions of gender‐typical characteristics (Charles & Grusky, 2004). It is conceivable that the presumption of different gender‐typical occupational aspirations impacted the female respondents' in the present study, leading the women to have higher educational motivation related to professional interests than their male counterparts. At the same time, the findings revealed few differences between men and women's educational motivations.

Age has been highlighted as a possible contributor to the differences in motivation among men and women; specifically, men seem to be older than women when they start their nursing education (Hoffart et al., 2019). More than half of the male respondents in the present study were over 24 years old, compared to just over a fifth of the women. Age had a significant impact on the *income and status* motivational factor in the present study; however, the students under the age of 24 years old had a significantly greater educational motivation for this factor.

The educational motivations of nursing students with immigrant backgrounds were primarily driven by income and status; this is in line with previous findings from another panel in the StudData database (Abrahamsen & Drange, 2014). We also investigated whether there were any differences in motivation driven by altruism or professional interests based on students' ethnicity; no significant differences were found in these areas.

Students with immigrant backgrounds reported a significantly higher motivation than the other students regarding the possibility of flexible work hours. One reason for this result may lie in the variable, “Good opportunities to balance work with family obligations,” which is part of the *anticipated time flexibility* factor (see Table 2). Basit (2012) found that young people with immigrant backgrounds viewed the opportunity to take care of their parents to be a key motivation to pursue higher education. At the same time, the study respondents may also interpret obligations toward family as caring for their own children. Regardless of which family members are the subject of such obligations, family unity is a key element in ethnic capital (Modood, 2004), and it is possible that the students with immigrant backgrounds emphasized this in their educational motivation.

### 5.2 Career expectations

The female nursing students in the present study had significantly higher expectations regarding prioritizing family life than the male nursing students. This was an expected result as women in nursing previously have expressed higher family‐related values (Ten Hoeve et al., 2016). The finding that male students had a higher expectation to work in management than female students was also expected since it has been documented in previous studies (Hoffart et al., 2019; Zysberg & Berry, 2005).

We also expected to find a difference between men and women in their expectations of pursuing a specialization. Nursing students have been shown to consider several fields of nursing to be better suited to women than men (McLaughlin et al., 2010; Muldoon & Reilly, 2003), and the majority of the most sought‐after positions in nursing (Kloster et al., 2007) require a specialization (Norwegian Directorate of Health, 2017). However, our findings revealed no differences between men and women's expectations of pursuing a specialization. Meanwhile, our study showed that male students expected to work in positions outside nursing even though they were only in their first year of study. This sentiment has been documented among male nursing students in their final year of study (Abrahamsen & Storvik, 2019).

In our study, age had no significance on respondents' career expectations. Therefore, it appears that gender is more important than age in terms of career expectations. Charles and Grusky (2004) claims that men and women are presumed to have fundamental different tastes and aptitudes. In this context, individuals make educational and professional choices, but do so in ways that reflect their own gendered preferences, and the social standards associated with gender‐appropriate work (Charles & Grusky, 2004). The fact that male and female nursing students to a large extent claim the same motivation for becoming nurses but different career expectations may be an expression of gender role‐related preferences.

Nursing is a profession with connections to the female gender role (Muldoon & Reilly, 2003; Myklebust, 2020). Occupations with higher proportions of women often pay less than occupations with higher proportions of men, and the incentives for both men and women to enter predominantly female occupations remains low (England, 2010). Inequalities of opportunity may be delegitimized within the different occupations in the labour market, yet it does not necessarily avoid individuals from seeing their own traits as either masculine or feminine. Even though Norway may be viewed as a modern egalitarian country, equal opportunities does not exclude the conception of men and women to have essential different attributes (Charles & Grusky, 2004). The fact that male students have career expectations that are not directly related to traditional nursing may relate to how inappropriate the practice of nursing is considered to be for men compared with other career opportunities. Meanwhile, the presumption of women to play a caring role could make it more natural for women to apply for a career where that role can be fulfilled. As men are not expected to have similar care functions, it becomes easier for them to pursue more socially accepted positions that provide opportunities for promotions and career progression (Charles & Grusky, 2004).

Respondents with immigrant backgrounds had lower expectations of pursuing a specialization than other students. If the demand for nurses in the labour market is high (Kalstø, 2019), this can make the pursuit of a specialization unfavourable. However, people with immigrant backgrounds have emphasized education as a way to achieve higher social mobility (Modood, 2004). It might be that students with immigrant backgrounds aim higher than other nursing students since the pursuit of high education and competence levels can act as a strategy to prevent discriminatory employment practices (Lee & Zhou, 2015).

Many of the specializations in Norway require a master's degree (Norwegian Directorate of Health, 2017) and students with immigrant backgrounds have stated high expectations to obtain higher education before (Abrahamsen & Drange, 2014). In the panel data, the respondents in the present study were not asked about their expectations of pursuing master's degree programmes. Abrahamsen and Drange (2014) have focused on students studying several different professional fields, and it may be that expectations of pursuing specializations and master's degree programmes can vary based on the educational programme (Abrahamsen & Drange, 2014).

Ethnic capital may also inhibit an individual's educational progression, and we cannot rule out that the respondents' were influenced by this when they completed the survey. For example, cultural gender differences may limit an individual's educational and career choices (Shah, 2007). Educational expectations may also lead young people with immigrant backgrounds to choose education programmes that they do not really want, which may affect their educational performance (Leirvik, 2016).

Compared with other nursing students, those with immigrant backgrounds had lower expectations of working as nurses in the future. Abrahamsen and Drange (2014) also reported that students with immigrant backgrounds had lower expectations of working in a position related to their education, while Orupabo (2014) found that nursing students in general are positive about their future employment. Meanwhile, Drange and Alecu (2019) posited that immigrants are less likely to be employed in a job that requires authorization, such as nursing. In addition, Orupabo (2014) found that nurses with immigrant backgrounds had limited access to positions in somatic and psychiatric hospitals.

Drange and Karlsen (2016) indicated that nurses with immigrant backgrounds are more likely to work in elderly care, which is among the least popular working fields among nursing students (Hunt et al., 2020; Kloster et al., 2007). However, seeking work in elderly care could be understood as a strategy to avoid discrimination in the labour market (Modood & Khattab, 2016). For example, nursing students with immigrant backgrounds may calibrate expectations or redefine their goals and apply for positions in elderly care in response to job opportunity limitations (Orupabo, 2014).

Discriminatory employment practices can also be a reality for nursing students with immigrant backgrounds (Midtbøen & Rogstad, 2012). Lower expectations of specializing and working as nurses may therefore be an expression of the impact of such barriers.

### 5.3 Limitations

This study examined the influence of gender and ethnicity on first‐year nursing students' educational motivation and career expectations. To our knowledge, this is a topic without a solid research basis. The StudData set offers suitable data for our purpose, but the insights need further validation to become conclusive evidence. Although we suggest carefulness, the items have been used in previous studies that have examined the motivation (Aamodt & Jensen, 2002; Nesje, 2014) and career expectations in nursing students (Abrahamsen, 2019; Abrahamsen & Drange, 2014). As the StudData survey has contributed to numerous publications,^3^ they were considered suitable for the analyses.

Another potential limitation in our study is the time since the data set was collected. Even though societal values and attitudes may have changed since 2012, the distribution of men and women in the nursing profession is quite stable (SSB, 2020a) and in nursing education there have only changed little (SSB, 2019). Therefore, we are confident that the data analysed here is still relevant. The proportion of nursing students with immigrant backgrounds increased from one‐tenth in 2012 to over 15% in 2019 (SSB, 2020b), but the distribution from the countries with the largest immigrant groups appears to be similar with the distribution in 2012 (SSB, 2021). An additional potential limitation is the abstract measure of age and ethnicity. The data processing agreement with SPS entailed an anonymization of the participants' age, and the variable was pre‐categorized into students over and under 24 years old. In addition, the respondents in the immigrant background category, as used within this paper, represented a heterogeneous group since we included many different continents of origin to make the category. Although the data set did not provide information on the countries of origin of the respondents in this category, it must be noted that the significance of ethnicity could vary based on the students' national and cultural backgrounds. Despite its limitations, the present study builds on and add to previous research findings that ethnicity and gender are important for individuals' career choices by emphasizing the importance of these factors when choosing nursing education.

The present study's cross‐sectional design was well suited to analyse a great deal of data on the topic. This type of design often provides data on complex relationships, and there may be unknown factors that affect the variables (Polit & Beck, 2017). Since the study focused on nursing students from three educational institutions in 2012, the sample is not representative of the entire population of 13,855 Norwegian nursing students in that year (SSB, 2020b). By bootstrapping the analyses, we prevented deviations by estimating the underlying distribution in the population. However, a disadvantage of bootstrapping is the uncertainty it creates in the confidence interval limits (Skovlund, 2019). The dependent variables were also measured at the ordinal level. This enabled a relative ranking of the individual values; however, we could not determine anything about the distance between each value, which, in turn, affected the significance of the mean of the dependent variables (Polit & Beck, 2017).

## 6 CONCLUSION

The present study examined gender and ethnicity in relation to nursing students' educational motivation and career expectations. The results showed that female and male nursing students had almost the same motivations to become nurses. However, female students were more driven by professional interests than male students, and there were clear differences between the women and men in their career expectations as nurses. Female nursing students had higher expectations of prioritizing time with family, while men had higher expectations of working in leadership positions and being employed in work outside of traditional nursing. The study findings also showed clear differences between nursing students with and without immigrant backgrounds. Students with immigrant backgrounds were more driven by extrinsic motivational factors, such as salary expectations, status, and flexible work hours, and had lower expectations to pursue a specialization or assume positions related to their education.

Norwegian authorities want to equalize educational fields that are overrepresented by one gender (Ministry of Children, Equality & Family, 2015) and facilitate equal employment opportunities regardless of gender and ethnicity (The Royal Norwegian Ministry of Finance, 2019). The present findings showed that nursing students' educational motivation and career expectations vary based on ethnicity and gender. Compared to previous findings, these trends seem to have a connection with the career paths observed among nurses with different gender and ethnicity. Furthermore, social presumptions of the differences in characteristics between men and women contributes to the maintenance of gender differences in the labour market, which would, in turn, lead students to narrow their choice of educational and professional alternatives within a normative context (Charles & Grusky, 2004).

Meanwhile, ethnic capital can facilitate choice of higher education (Modood, 2004), while labour market barriers may lead to lower career expectations among students with immigrant backgrounds (Midtbøen & Rogstad, 2012). To address gender distribution and promote diversity in nursing, nursing educational institutions and the nursing profession must promote a labour market in which nurses' competence and skills are sought‐after, assessed, regardless of their gender and ethnicity. Nursing educational institutions need to be aware that student recruitment is influenced by gender role‐related differences between men and women. The health service needs to recognize that graduates with immigrant backgrounds may face obstacles in the labour market that are unrelated to their competence. Future research could explore ethnic and gender differences in nursing with an emphasis on how these factors affect and reinforce each other, and how they affect nurses' career choices and career expectations.

## CONFLICT OF INTEREST

The authors have no conflicts of interest to declare.

## AUTHOR CONTRIBUTIONS

SM analysed, while SM, EB and AM interpreted the data regarding the nursing students' educational motivation and career expectations. SM was a major contributor in writing the manuscript. All authors read and approved the final manuscript.

## Data Availability

The data that support the findings of this study are available from the Centre for the Study of the Professions (SPS) at Oslo Metropolitan University. Restrictions apply to the availability of these data, which were used under license for this study. Data are available at https://www.oslomet.no/en/about/sps/our‐research/studdata with the permission of SPS.

## ENDNOTES

^1^
For more information, see https://www.oslomet.no/en/about/sps/our‐research/studdata

^2^
The questionnaire is available upon request.

^3^

http://nsddata.nsd.uib.no/webview/index/en/Individdata/Andre‐klassifiseringer.d.243/Kronologisk‐oversikt‐over‐datasett.d.36/2005.d.145/StudData‐Database‐for‐studier‐av‐rekruttering‐og‐kvalifisering‐til‐profesjonell‐yrkesutoeving‐Panel‐3/fStudy/NSD1338‐3
